# Glucocorticoid treatment and adrenal suppression in children: current view and open issues

**DOI:** 10.1007/s40618-024-02461-9

**Published:** 2024-10-01

**Authors:** Nicola Improda, Laura Chioma, Donatella Capalbo, Carla Bizzarri, Mariacarolina Salerno

**Affiliations:** 1 Neuro-Endocrine Diseases and Obesity Unit, Department of Neurosciences, Santobono- Pausilipon Children’s Hospital, Napoli, Italy; 2 Endocrinology Unit, University Hospital Pediatric Department, Bambino Gesù Children’s Hospital, Rome, Italy; 3 Pediatric Endocrinology Unit, Department of Translational Medical Sciences, University of Naples Federico II, Endo-ERN Center for Rare Endocrine Conditions, Naples, Italy

**Keywords:** Glucocorticoid-induced adrenal insufficiency, Adrenal crisis, Adrenal suppression, Steroid treatment, Glucocorticoid replacement

## Abstract

**Purpose:**

Glucocorticoids (GCs) are commonly used for several acute and chronic pediatric diseases. However, chronic treatment may result in hypothalamic-pituitary-adrenal axis (HPA) dysfunction. Glucocorticoid-induced adrenal insufficiency (GI-AI) is indeed the most frequent cause of adrenal insufficiency (AI) in children, possibly resulting in a life-threatening event such as adrenal crisis (AC). It is generally underestimated, especially when using non-systemic glucocorticoid formulations. This review aims at summarizing current evidence on the effects of long-term GC treatment on the HPA axis, management of GC tapering and assessment of the HPA recovery.

**Methods:**

We conducted a narrative review of the relevant literature focusing on pathogenic mechanisms, predictive factors, diagnosis and treatment of GI-AI.

**Results:**

All types of GCs, whatever the route of administration, may have suppressive effects on the HPA axis, especially when compounds with higher potency and long half-life are used. Moreover, chronic GC administration is the most common cause of Cushing syndrome in children. In order to overcome the risk of GI-AI, slow withdrawal of GCs is necessary. When approaching the replacement dose, it is recommended to switch to shorter half-life formulations such as hydrocortisone. Assessment of HPA axis recovery with basal and stimulated cortisol levels may help detecting children at risk of AC that may require hydrocortisone supplementation.

**Conclusion:**

The management of GI-AI in children is challenging and many areas of uncertainty remain. Improving the knowledge on long-term GC effects on HPA in children, the management of steroid discontinuation and emergency dosing may help preventing GI-AI symptoms and acute hospital admission for AC.

## Introduction

Glucocorticoids (GCs) represent the cornerstone of the treatment of inflammatory, or immune-mediated diseases and malignancies in children. It has been estimated that approximately 1% of the adult population is chronically exposed to oral GCs, most frequently prescribed for respiratory diseases [1, 2]. Data regarding GC exposure in childhood are scanty, however it is well known that most pediatricians, during their career, will prescribe steroids to children and/or will manage children on GC therapy.

**Fig. 1:**
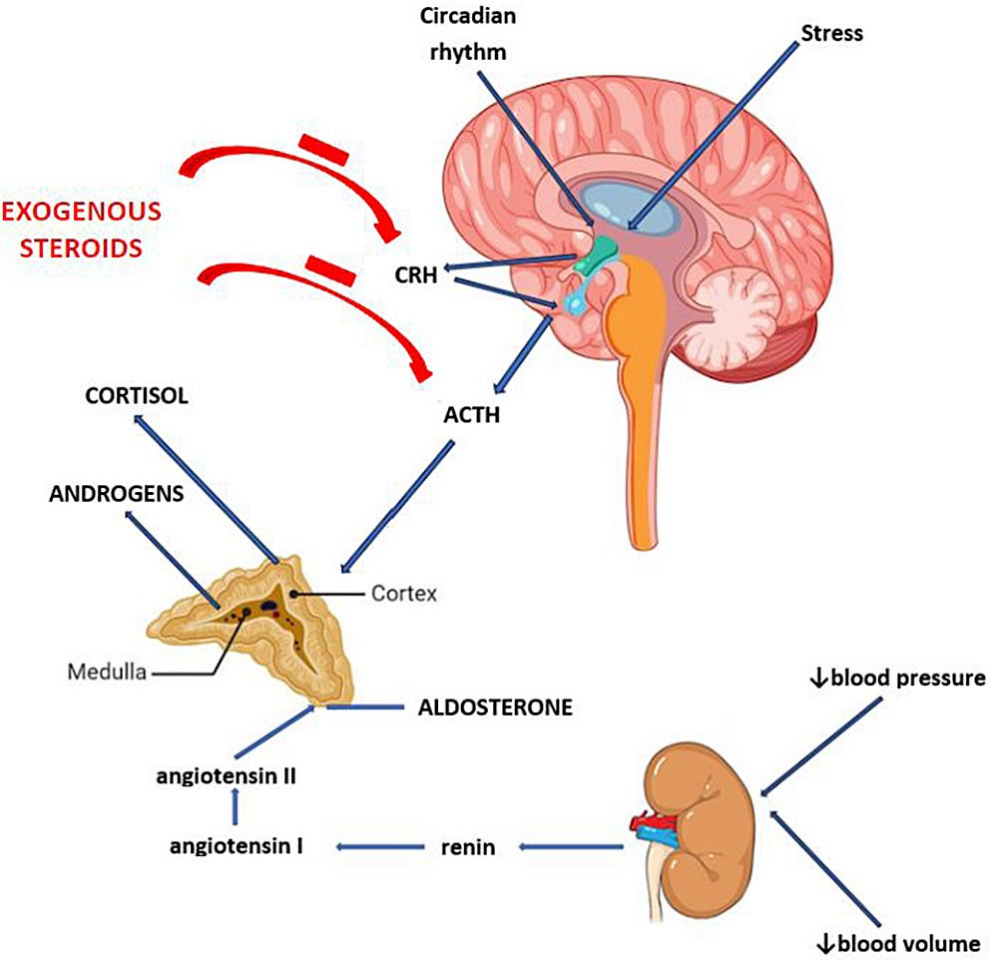
Pathophysiology of glucocorticoid-induced adrenal insufficiency

Prolonged exposure to GCs leads to a negative feedback on the release of both corticotropin-releasing hormone (CRH) and adrenocorticotropic hormone (ACTH), with subsequent impaired secretion of cortisol from the adrenal gland, which can persist after discontinuation of GCs for a variable duration [3–6] (Fig. 1). This condition, also referred to as GC-induced adrenal insufficiency (GI-AI) is the most common form of tertiary adrenal insufficiency (TAI) in children.

Adrenal insufficiency (AI) is a condition characterized by inadequate adrenal cortisol production, and can be primary, secondary, or tertiary according to the underlying mechanism.

Primary AI (PAI) is a chronic condition due to abnormalities of steroid biosynthesis or of adrenal gland development and responsiveness. It is characterized by impaired secretion of GCs and can be accompanied by mineralocorticoid and adrenal androgens deficiency or excess, depending on the underlying cause [7].

Secondary AI (SAI) results from any process that involves the pituitary or hypothalamus and interferes with adrenocorticotropic hormone (ACTH) secretion such as congenital, malformative, genetic syndromes as well as several acquired conditions of different etiologies. It may be isolated or associated with other pituitary hormone deficiencies [8, 9].

TAI is caused by factors affecting the hypothalamic region that result in reduced corticotropin-releasing hormone (CRH) secretion, such as tumors, radiation, inflammatory processes ore most commonly by suppression of the hypothalamic-pituitary-adrenal (HPA) axis by chronic administration of high doses of GCs [5, 8, 10, 11]. A similar form of TAI occurs in patients successfully treated for Cushing syndrome, due to prolonged exposure to excess endogenous GCs [5, 12].

Differentiation between SAI and TAI is often difficult and mixed forms may occur especially in those cases secondary to medications [8]. Both conditions are characterized by the lack of ACTH stimulation resulting in reduced cortisol secretion and thus the term central AI (CAI) is used without further differentiation between the two entities [5]. Secretion of aldosterone is preserved in CAI because it is mainly regulated by the angiotensin/renin system and only marginally affected by ACTH levels [5].

The risk of GI-AI is generally under-estimated, especially when GCs are administered by routes different from systemic, i.e. inhalatory or topical. This is partly because the condition is perceived as transient and AI symptoms are generally non-specific, overlapping with those of the relapsing underlying disease, as well as those of the so-called GC withdrawal syndrome (GWS) [13].

Systematic reviews and meta-analyses in adults indicate percentages of patients with GI-AI ranging between 1.4 and 60%, based on the administration form, dosing, and treatment duration of GCs [3, 14]. A large variability of results exists between studies, depending upon sample size, frequency or duration of tapering and methods of AI assessment in terms of diagnostic test and cut-off used [6]. Although the risk of AI decreases over time, 15% of the subjects still have the condition 3 years after GC discontinuation [14].

Studies in children report a prevalence of GI-AI between 31 and 60% in patients on physiological replacement therapy, tested at least one month after discontinuation of treatment [3, 15, 16]. Data from the Canadian Paediatric Surveillance Program, a survey involving over 2500 pediatricians, reported an annual incidence of symptomatic GI-AI in children aged 0–18 years equal to 0.35/100 000 [13]. Sixty-five per cent of the children experiencing GI-AI were treated for asthma, and 80% were exposed to inhalatory steroids, alone or in combination with other formulations. Moreover, in children with inflammatory bowel diseases (IBD) the prevalence of adrenal suppression after gradual tapering of steroids is estimated at about 20% [17].

In order to overcome the risk of GI-AI, slow withdrawal of GC over several months is necessary to warrant full recovery of the HPA axis [4, 5, 18]. Nonetheless, there is no consensus regarding the optimal GC tapering protocol in childhood across different centers and different diseases. Moreover, it remains debated whether and when recovery of adrenal axis function should be tested.

The diagnosis of AI is challenging and the majority of patients are only diagnosed during an acute hospital admission [8, 19, 20]. Indeed, in subjects with GI-AI intercurrent stressful events and/or abrupt discontinuation of GC treatment may result in a life threatening adrenal crisis (AC) [6, 12, 13, 20].

Approximately 6 to 24% of adults with AI manifest an episode of AC each year. In children with AI (both PAI and CAI), the frequency of AC is reported to be 3.4 per 100 patient-years [21] with about 1/200 cases of AC being fatal [20]. AC is more frequent in patients with PAI than in those with CAI due to partially preserved cortisol secretion and intact mineralcorticoid production in the latter forms [22, 23].

Finally, inadequate education on management of GI-AI is reported as well as reduced likelihood to receive proper care in the emergency setting in comparison to AI from other etiologies, thus suggesting a lack of awareness of this specific condition [11, 22].

The purpose of this review is to consolidate existing knowledge about GI-AI, in order to ameliorate awareness regarding the condition and provide healthcare professionals with theoretical and practical suggestions to improve management of GC treatment, and minimize AI-related morbidity and mortality in childhood.

## Glucocorticoid use in children

All the GCs have been synthetically developed from cortisol. Hydrocortisone is a synthetic compound molecularly identical to the endogenous cortisol. In general, receptor affinity, potency and anti-inflammatory/immunosuppressive power of the other synthetic GCs are greater than those of endogenous cortisol, with the only exception of cortisone acetate which is a prodrug with a slightly delayed onset of action because it needs to be activated into cortisol by the hepatic 11β-hydroxysteroid dehydrogenase enzyme (Table 1). Thus, only hydrocortisone and cortisone acetate should be used for replacement therapy in children affected with AI, allowing avoidance of side effects of other GCs, especially reduced linear growth, that is the most commonly seen side effect.

**Table 1:** Properties and biological potency of corticosteroid compounds

	Equivalent dose (mg)	Glucocorticoid activity	Mineralocorticoid activity	Relative anti-inflammatory potency	Half-life (hours)	Duration of action	Route of administration
Hydrocortisone	20	1	1	1	8–12	Short	Oral Parenteral Topical
Cortisone acetate	25	0.8	0.8	0.8	8–12	Short	Oral
Deflazacort	6-7.5	3	1	2.6	< 12	Short	Oral
Prednisone	5	4	0.8	4	12–36	Intermediate	Oral
Prednisolone	5	4	0.8	4	12–36	Intermediate	Oral Parenteral Topical
Triamcinolone	4	5	0	5	12–36	Intermediate	Oral Parenteral Topical
Methylprednisolone	4	5	0.5	5	36–72	Long	Oral Parenteral Topical
Dexamethasone	0.75	25	0	30	36–72	Long	Oral Parenteral Topical
Betamethasone	0.8	25	0	25	36–72	Long	Oral Parenteral Topical

The daily physiological secretion of cortisol by the adrenal gland has been estimated to be 5–10 mg/m² surface area [24, 25]. Hence, a dose of 8–10 mg/m² is suitable in children for cortisol replacement in unstressed conditions [24, 25]. Higher doses (10–15 mg/m² surface area) are necessary in congenital adrenal hyperplasia, with the additional aim of suppressing adrenal androgen excess [26]. Cortisol secretion increases many-fold in response to physical and emotional stress, proportionally to the severity of the stress [27, 28], and thus in subjects with AI the dose of hydrocortisone must be increased in stressful conditions, aiming at mimicking physiology.

GCs are also used to treat relative adrenal insufficiency in patients with serious illness experiencing refractory hypotension despite adequate inotropes and crystalloids administration. This condition, also known as critical-illness-related corticosteroid insufficiency (CIRCI), is characterized by resistance to endogenous cortisol and/or secretion of cortisol that is inappropriate for the severity of the disease [29].

Besides replacement therapy, GCs have important anti-inflammatory and immunosuppressive properties (Table 1), via both genomic and non-genomic actions [30]. Nationwide studies showed that approximately 1% of adults are chronically treated with GCs [1, 2]. Indeed, systemic (oral or parenteral) GCs administered in supraphysiological doses are essential components of treatment protocols for several disorders related to immune response dysfunction, such as glomerulonephritis [31], rheumatic diseases [32], inflammatory bowel diseases [33, 34], autoimmune hepatitis [35], acute leukemia [36], and graft-versus-host disease (GvHD) [37]. In some of these conditions alternate day administration, or pulse therapy with high dose systemic GCs, have been conceived with the specific aim of reducing the incidence of GI-AI [38].

Non-systemic GCs are widely used in children. Inhalatory GCs are most often prescribed for asthma [39], while intranasal GCs through an inhaler or nebulizer are used for the treatment of allergic rhinitis, rhinosinusitis, rhinoconjunctivitis, and nasal polyposis [40]. Transcutaneous GCs are the mainstay of treatment of several skin disorders, including atopic dermatitis, vitiligo, and psoriasis [41]. Intraarticular injection of GCs, such as triamcinolone and methylprednisolone, is largely used to induce fast remission of monoarticular juvenile idiopathic arthritis [32]. Rectal GCs are administered in patients with inflammatory bowel diseases affecting the lower part of the colon and rectum to limit systemic exposure [33, 34]. In general, short term use is considered safe, even if systemic absorption has been demonstrated [13, 14, 22, 42, 43].

Supraphysiological doses of GCs may exert detrimental effects, as impaired linear growth, obesity, osteoporosis, myopathy, glaucoma, cataract, increased risk of infections, altered glucose homeostasis, and behavior disturbances. Moreover, prolonged GC administration is the most common cause of Cushing syndrome in children, with an incidence influenced by both the dosage and length of treatment [42, 44].

Whatever the administration route, HPA axis suppression is the most serious adverse effect of GCs as, albeit rarely, it may result in prolonged AI and life-threatening AC [6, 13, 14, 22, 43].

Although non-systemic administration of GCs was initially thought to avoid systemic side effects, there is now clear evidence that all types of topical GCs (inhaled, intranasal, intra-articular, transcutaneous) have a significant bioavailability in the systemic circulation, with reproducible suppressive effects on the HPA axis, especially when compounds with higher potency and long half-life are used [45–49].

Moreover, topical GCs may exert formulation-specific local side effects (i.e. skin atrophy and/or striae for transcutaneous GCs, cataract and glaucoma for ophthalmic forms, and oral candidiasis for inhaled steroids) that may limit their use in clinical practice [45, 50].

Systemic bioavailability of transcutaneous steroids largely depends upon the compound used and the characteristics of the pharmaceutical formulation. Additionally, the presence of cutaneous inflammation, impaired barrier function due to skin damage, occlusive dressings, and the site of application can affect systemic absorption [51]. Indeed, absorption is higher through skin areas as eyelids and diaper regions in infants and through mucous membranes [50].

Inhaled GCs can reach the general circulation through different ways. A proportion variable from 10 to 20% is directly inhaled and reaches the lungs unaltered, while up to 90% is deposited in the mouth and pharynx and then swallowed, reaching the gastrointestinal tract where it can be absorbed [49]. Of note, the use of a spacer can reduce the proportion of inhaled GCs that is swallowed up to 10% [49].

The proportion that survives the first pass liver metabolism can exert systemic effects. Such properties of inhaled GCs is defined as oral bioavailability and largely depends upon the compound used, being low (around 1%) for ciclesonide and fluticasone, medium (around 10%) for budesonide and maximum (20–40%) for beclomethasone [49, 52]. Most of the reported AI cases in children treated with inhaled GCs have been linked to the use of fluticasone propionate [6, 52, 53]. Indeed, despite low oral bioavailability, fluticasone propionate has several other pharmacodynamic and pharmacokinetic properties that favor the development of adrenal suppression, such as high degree of systemic tissue retention, prolonged half-life (far superior to other inhalatory steroids), low degree of degree of plasma protein-binding, and strong binding affinity for the glucocorticoid receptor (around 18 times that of dexamethasone) [49, 52, 53].

## Clinical issues related to steroid withdrawal

GI-AI represents the most common tertiary form of AI. Chronic GC treatment leads to a reduction in CRH production by the hypothalamus and ACTH production by the pituitary (Fig. 1). Over time this can result in adrenal atrophy. After discontinuation of therapeutic/supraphysiological doses of GCs, the secretion of ACTH is the first to be restored, followed by recovery of CRH and eventually cortisol and androgens secretion. However, when adrenal atrophy occurs, abnormal cortisol and/or androgen secretion may persist for a long time [54].

In children who are unable to produce an appropriate cortisol response, exposure to an acute stressor, as an intercurrent illness, can lead to symptoms of adrenal insufficiency and potentially result in adrenal crisis [43, 55]. The risk of symptomatic AI increases when the daily dose of GCs becomes lower than the equivalent substitutive dose, or when GCs are abruptly discontinued [13, 56, 57]. Data investigating the risk of mortality associated with GI-AI in children are currently lacking. However, a recent study in adults based on national registry data reported that GI-AI may likely contribute to death in 3.7% of the patients taking oral GC who had died in hospital [58].

Clinical features of GI-AI are detailed in Table 2. GI-AI is the only form of AI that may coexist with Cushing’s syndrome in the same patient for some time, after the complete discontinuation of GC therapy. Other symptoms are generally nonspecific. Poor linear growth has been reported in up to 50% of children with GI-AI [13]. It must be noted that, in addition to being related to GC therapy itself, growth failure is also a sign of iatrogenic AI [42]. As far as weight is concerned, during the time of chronic steroid use infants may have poor weight gain, while older children and adolescents more commonly exhibit excess weight increase, with possible development of central obesity [9, 42].

**Table 2:** Clinical features of glucocorticoid-induced adrenal insufficiency

Signs/symptoms	Iatrogenic Adrenal Insufficiency	Iatrogenic Cushing	Adrenal crisis
General aspect	Feeling generally unwell Pale/alabaster-like skin	Moon-like face Facial plethora Supraclavicular and dorsocervical fat pads Acne Peripheral edema Hypertrichosis Striae rubrae Easy bruising Skin atrophy	Fever Unconsciousness Collapse
Growth	Poor weight gain associated with reduced linear growth (mostly infants) Reduced linear growth with normal weight gain (late childhood and adolescence) Weight loss (mainly due to gastrointestinal symptoms)	Central obesity with reduced linear growth	Weight loss (dehydration)
Gastro-intestinal	Anorexia Nausea/vomiting Abdominal discomfort	Increased appetite	Nausea/vomiting Abdominal pain (possibly mimicking surgical conditions)
Neurological	Dizziness (mainly postural) Headache Somnolence Confusion	Emotional lability Depression Insomnia Pseudotumor cerebri	Lethargy Confusion Delirium Coma Seizures
Musculoskeletal	Myalgia/arthralgia (especially hands) Weakness/fatigue	Proximal muscle weakness	Severe fatigue
Other	Postural hypotension Hypoglycemia Hyponatremia Lymphocytosis and eosinophilia	Hypertension Hyperglycemia Poor wound healing Menstrual irregularities	Hypotension up to hypovolemic shock Hypoglycemia Hyponatremia

Glucocorticoid withdrawal syndrome (GWS) represents a manifestation of dependence on high concentrations of GC [59]. It may occur when the dose is still supraphysiological, but lower than the usual dose and may even persist after the response of the HPA axis to stimuli is restored [60]. Despite being reported in up to 67% of adults [61], this condition is seldom described in childhood [62]. GWS is thought to be multifactorial, being mediated by inhibition of central noradrenergic and dopaminergic networks, decrease in CRH and POMC-related peptides acting as endogenous opioids, and increased levels of cytokines (interleukin 6, tumor necrosis factor α) and prostaglandins [59, 63]. GWS is self-limited, but may last months, thus impacting negatively on the patient’s quality of life. GWS symptoms may overlap with symptomatic AI and/or with a relapse of the underlying disorder, including anorexia, nausea/vomiting, lethargy, arthralgia, myalgia, low grade fever, postural hypotension, and psychiatric symptoms such as anxiety, panic attacks and depression [59]. Of note, under-recognition of this condition as a separate entity may lead to unnecessary increase in GC doses making recovery of the HPA axis more delayed.

## Predictive and predisposing factors for GI-AI

Given the burden of GI-AI for both the families and the health-care system, several studies [3, 4, 15] have attempted to find factors that may predict its occurrence, in order to establish preventive strategies. Table 3 summarizes the main factors predicting higher risk of GI-AI.

**Table 3:** Factors predicting higher risk of glucocorticoid-induced adrenal insufficiency

**Absolute risk factors** • Duration of treatment > 2 weeks • ≥ 3 cumulative weeks of systemic glucocorticoids in the last 6 months • ≥ 3 months of high doses inhalatory glucocorticoids§ • Symptoms of AI while on replacement doses, or after discontinuation of glucocorticoids • Growth failure	
**Duration-dependent additional risk factors** • Concomitant use of other drugs that modify GC metabolism¶ • High daily or cumulative supraphysiological dose of glucocorticoids • Corticosteroids with high tissue half life • Oral or intra-articular formulations • Multiple routes of administration • Daily or multiple daily doses • Evening doses	

§ ≥400–500 µg/day of fluticasone or ≥ 800–1000 µg/day of budesonide or beclomethasone or ≥ 1000 µg/day of ciclesonide

**¶CYP3A4 inhibitors - Moderate**: amiodarone, aprepitant, cimetidine, conivaptan, crizotinib, ciclosporin, diltiazem, dronedarone, erythromycin, fluconazole, fosamprenavir, fosaprepitant, grapefruit juice, imatinib, isavuconazole, netupitant, nilotinib, ribociclib, schisandra, and verapamil; **Strong**: boceprevir, ceritinib, clarithromycin, cobicistat, darunavir, idelalisib, indinavir, itraconazole, ketoconazole, lopinavir, mifepristone, nefazodone, nelfinavir, posaconazole, ritonavir, saquinavir, telaprevir, telithromycin, and voriconazole;

**CYP3A4 inducers - Moderate**: Bexarotene, Bosentan, Dabrafenib, Dexamethasone, Efavirenz, Eslicarbazepine, Etravirine, Modafinil, Nafcillin, Rifabutin, Rifapentine, St. John’s wort; **Strong**: Carbamazepine, Enzalutamide, Fosphenytoin, Lumacaftor, Mitotane, Phenobarbital, Phenytoin, Primidone, Rifampin (rifampicin)

It is well known that higher doses (both daily or cumulative) and longer durations of treatment are associated with the highest risk of having glucocorticoid-induced AI [3]. However, these factors do not allow a reliable prediction in all cases [3, 64]. In a recent metaanalysis of studies in adults [3], even short-term (< 1 month) treatment or doses below the recommended range have been associated with a not negligible risk of AI, and there is evidence demonstrating histological and functional changes in the adrenal cortex even after administration of steroids for just 5 days [65]. Multiple short courses of GC therapy can also cause adrenal suppression [66]. Even in children GI-AI does not seem to be correlated with treatment duration, age at treatment initiation, or cumulative GC doses [15, 16].

Pharmacokinetic and pharmacodynamic properties of GCs play an important role in the development of GI-AI. Indeed, the use of GCs with longer tissue half-life (> 48 h), such as dexamethasone and betamethasone, makes GI-AI more likely (Table 1). On the other hand, prednisone, prednisolone, methylprednisolone and triamcinolone are moderately suppressive, while hydrocortisone, cortisone acetate and deflazacort exert the least suppressive effect [67] (Table 1). Even though all the formulations of GCs available may result in AI, intra-articular, oral, and multiple formulations are associated with the higher risk (about 50%), while topical or intranasal routes may result in a lower risk (about 5%) [3, 68, 69]. Table 4 summarizes risk factors other than the dose specific for topical GCs.

**Table 4:** Factors additional to the dose that increase the risk of glucocorticoid-induced adrenal insufficiency when using topical glucocorticoids

**Inhaled glucocorticoids** • Administration > 3–6 months • Treatment with fluticasone propionate • No step-down approach in asthma (active disease and airflow obstruction increase absorption) • Concomitant use of oral or intranasal glucocorticoids (including intermittent use) • Lower body mass index • Optimal compliance to treatment	
**Intra-articular glucocorticoids** • Repeated injections • Inflammatory arthropathies (increased absorption)	
**Percutaneous glucocorticoids** • Use of glucocorticoids with higher potency • Prolonged use on skin inflamed or with impaired barrier function • Occlusive dressings • Use on mucous membranes, eyelids, and scrotum • Larger body surface area to body weight ratio (especially infants)	

In asthmatic children, up to two-third of subjects receiving inhalatory GCs for at least 2 months exhibited some degree of HPA axis dysfunctions, and about one third had persistent adrenal suppression [70, 71]. In a recent meta-analysis including 522 children from 2 studies, 3.8% had biochemically documented AI, almost exclusively treated with high-potency GCs [72]. Indeed, the risk of GI-AI with inhalatory GCs depends upon the drug used and the duration of treatment (higher risk for beclomethasone and fluticasone, lower risk for ciclesonide and budesonide), and is significantly higher in patients who are concurrently treated with oral and/or intranasal GCs and/or present with blunted growth [3, 50, 70, 73, 74].

The choice of the inhalation device and particle size largely influence oral deposition and drug delivery to the lung [74]. Despite a general lack of studies comparing adrenal suppression among different types of inhalers [75–78], it has been recommended to keep lower doses when using devices associated with greater lung delivery [73]. According to recent recommendations, all children who have been treated for at least 3–6 months with ≥ 400–500 µg/day of fluticasone or ≥ 800–1000 µg/day of budesonide or beclomethasone or ≥ 1000 µg/day of ciclesonide should be screened for AI [13, 50, 78, 79].

Fluticasone furoate has increased retention time in the airways [80] with less relevant systemic bioavailability and negative impact on the HPA axis, compared to fluticasone propionate [80, 81]. Budesonide, a GC with predominantly local action, has also been studied in several pediatric gastrointestinal diseases, aiming at reducing the risk of GI-AI. In the case of eosinophilic esophagitis normal adrenal function was documented in most [82, 83] but not all [84] studies involving subjects chronically treated with oral viscous budesonide. Moreover, enteric-coated budesonide has been shown to cause adrenal suppression in children with Crohn’s disease, even though to a lesser extent (about one third of the patients) than prednisone or prednisolone [85, 86]. Rectal formulations of GCs, like beclomethasone dipropionate, have been designed to act topically in the site of inflammation, with reduced bioavailability and risk of GI-AI than systemic GCs [87, 88].

Concomitant use of other drugs may increase biologic half-life of GCs and enhance the adrenal-suppressant effects of GCs, mainly by inhibiting cytochrome P450 3A4 (CYP3A4) enzymes (Table 3), responsible for their metabolic clearance [57, 81]. Therefore, concomitant treatment with strong CYP3A4 inhibitors and inhaled, intranasal, and injectable fluticasone, budesonide, and triamcinolone is not recommended (89–90). In this regard, the results of a recent Cochrane review indicated that children with acute leukemia taking antifungal therapy may display more prolonged AI, in a dose-dependent fashion [57]. Moreover, concomitant treatment with ritonavir leads to an increase in the circulating concentrations of the active GC metabolites up to 37% [91], resulting in high risk of iatrogenic Cushing’s syndrome and symptomatic AI, even when using inhaled and intranasal GC formulations. This is particularly relevant in HIV infected patients [91].

Conversely, the addition of drugs that enhance GC metabolism may cause latent adrenal insufficiency to become symptomatic, especially when the patient is on physiological replacement doses [4] (Table 3).

Timing of GC administration also seems to play an important role. Evening doses result in a higher risk of developing GI-AI, compared to morning doses, because of higher sensitivity to GCs at that time [92] and suppression of the early-morning ACTH peak, leading to a profound disruption of the physiological circadian rhythm [92].

Multiple doses may also account for a higher risk of GI-AI than single daily doses, due to higher and more constant 24-hour exposure to glucocorticoids [93]. Gene expression in circulating blood cells of healthy subjects receiving exogenous GCs administered in multiple daily doses showed an abnormal circadian rhythmicity that partially normalized after switching to once a day administration [94].

Based on these predictive factors there has been a recent attempt to categorize GI-AI into arbitrarily defined very high, high, moderate, and low risk classes [4]. However, within the pediatric population, making a clear distinction between many different categories is challenging for several reasons. Firstly, the studies available on this topic are often limited by heterogeneous age and methods for assessment of adrenal function, use of concomitant drugs and multiple steroid formulations. Secondly, reliability of the predictive factors related to the treatment or the underlying condition is unpredictably hampered by the presence of individual predisposing factors. The sensitivity of the HPA axis to exogenous glucocorticoids has been demonstrated to be variable with relevant inter-individual variability of the dose-response relationship [6, 92]. Gene variants or single nucleotide polymorphisms of GC and mineralocorticoid receptors [95, 96], platelet derived growth factor D (PDGF) [97], other receptors involved in the regulation of the HPA axis reactivity [98], or enzymes that metabolize exogenous GC [99] can modulate the patient’s vulnerability to develop side effects of GCs. Finally, several constitutional factors, such as sex, age, and pubertal status may influence the suppressive effects of GCs on the HPA axis [100]. Indeed, adrenal suppression after transcutaneous GCs is more common in infants and toddlers, than in older children, due to an increased ratio between body surface area and body weight [101]. Moreover, an inverse correlation between stimulated peak cortisol and age has been found [101, 102].

Therefore, current data in children allow to identify only patients at higher risk to develop GI-AI, based on the presence of specific risk factors (Table 3).

## Glucocorticoid tapering

GCs should be discontinued when the maximum therapeutic benefit has been achieved or the patient has developed relevant side effects [4, 9, 42].

Although duration of treatment does not predict GI-AI in all cases, it is generally accepted that children taking GCs for less than 2 weeks, for example for allergic reactions or acute respiratory diseases, can discontinue therapy abruptly [4, 9, 42].

In conditions requiring long-term (> 2 weeks) supraphysiological doses of GCs, especially when cushingoid features are associated, gradual tapering of GC therapy has become routine practice, with the main aim of avoiding relapse of the underlying condition, while minimizing the risk of GI-AI and GWS [4, 9, 42, 103]. It must be noted that children who require frequent courses of GCs, like asthmatic or allergic subjects, must also be considered at higher risk [4]. On the contrary, the use of well spaced steroid pulses, like weekly iv methylprednisolone pulses [104] or monthly 4-day courses of dexamethasone [105], seems to be associated with a lower risk of long-term adrenal suppression.

GCs should be tapered at a rate established to maintain remission of the underlying condition. Therefore, a universal withdrawal protocol seems not conceivable, and disease-specific tapering protocols have been often suggested by the scientific societies, even if often based on low quality evidence [31, 33, 34]. In the therapeutic management of inflammatory bowel diseases [33, 34], a gradual decrease of prednisone/prednisolone equivalent to 10–20% every one-to-three weeks, in a single or divided doses, is suggested over 8 to 10 weeks. In the case of nephrotic syndrome, systemic lupus erythematosus, and other diseases, an alternate-day regimen for 6 weeks without any tapering is preferred [31]. Nevertheless, guidelines for specific pediatric conditions do not always provide explicit recommendations regarding assessment of the HPA axis recovery or they simply suggest evaluating morning cortisol concentrations in the presence of symptoms of AI [33, 34].

When the rate of tapering to prevent disease relapse is not specifically indicated, the possibility of increasing the risk of GI-AI through unnecessary prolonged taper should be considered [9]. In patients treated with chronic prednisone or prednisolone, especially in the presence of factors predicting high risk of AI, a reasonable approach consists in a stable weekly or bi-weekly reduction of 10–20% of the daily dose, with thorough clinical monitoring [9, 103]. During tapering, single morning doses of GCs should be preferred [4]. When the physiological dose has been reached (about 8 mg/m2/day of hydrocortisone equivalent), this should be kept in order to prevent symptoms of AI, and GCs can be eventually weaned off only if recovery of the HPA axis is proven. If treatment with long-acting GCs is no longer needed it is advisable to change to shorter half-life formulations such as hydrocortisone, which exerts a lower suppressant effect on the HPA axis, while waiting for full recovery of the HPA axis [106].

## Who needs to be tested for recovery of the HPA axis and how

Evaluation of the HPA axis recovery is recommended when the physiological dose has been reached or soon after discontinuation of GCs in all children who have been treated with systemic GCs for more than 2 weeks, or have at least one of the other factors predicting high risk of GI-AI (Table 3). Of note, testing for HPA axis recovery is useless if GCs cannot be discontinued, as for autoimmune hepatitis or organ transplants.

Even at physiological doses, exogenous GCs can interfere with HPA axis evaluation and should be temporarily discontinued shortly before testing. Discontinuation for 24 h is considered adequate for short-acting GCs (hydrocortisone), while longer discontinuation would be more suitable for intermediate and long-acting GCs (48 h for prednisone, 72 h for dexamethasone) [9, 107].

**Fig. 2:**
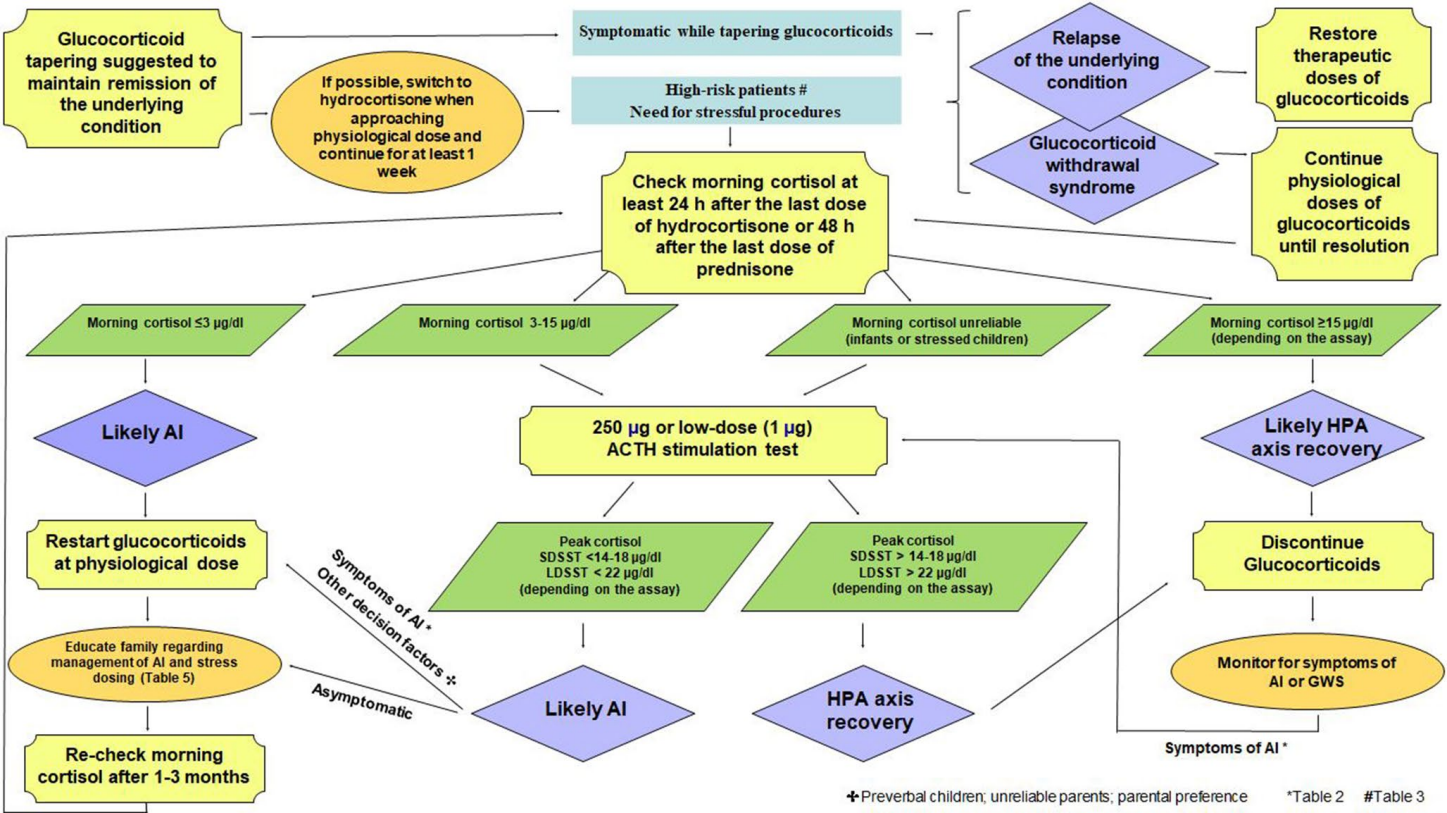
Management of glucocorticoid withdrawal and assessment of adrenal axis recovery

Figure 2 summarizes a practical approach to manage glucocorticoid withdrawal and to assess the recovery of adrenal function.

The measurement of morning fasting serum cortisol concentrations (between 7:00 and 09:00 AM) should represent the first step for the initial assessment of HPA axis integrity. However, accurate interpretation of cortisol concentrations can be challenging. Morning cortisol concentrations vary according to the individual circadian rhythm and assays used for measurements [4, 108] and the cut-off for morning cortisol that can reliably detect or rule out AI in children is still a matter of debate [15, 16].

A morning serum cortisol ≤ 3 µg/dl (83 nmol/L) in combination with low-normal ACTH was found to be specific for CAI [8, 106, 107, 109–111], while morning cortisol values > 16–18 µg/dl (450–500 nmol/L), depending on the assay, rules out CAI [8, 9, 108, 109, 112].

If morning cortisol suggests AI (< 3 µg/dl, 83 nmol/L) replacement GC therapy should restart with or switched to replacement hydrocortisone 8–10 mg/m2/day until the next evaluation of morning cortisol [9, 109].

If morning cortisol concentrations range between 3 and 15 µg/dl (83 and 420 nmol/L) further investigations, such as ACTH stimulation test, are needed to assess the stress responsiveness of the HPA axis [110]. Of note, values between 13 and 15 µg/dl (360–420 nmol/L) have been suggested to predict normal HPA axis function [9, 109, 112].

Several stimulation tests are available to confirm the diagnosis of GI-AI and may be also helpful in predicting the chance of future recovery of adrenal function.

Both standard-dose (250 µg) and low-dose (1 µg) ACTH (also referred to as cosyntropin or tetracosactide) stimulation tests are used in clinical practice, but a significant discussion about which test is superior to detect CAI is still ongoing [112, 113]. It has been shown that the low-dose Synachten test (LDSST) is more sensitive for the evaluation of CAI in children, with results more concordant with those of the insulin tolerance test, compared to the standard-dose Synachten test (SDSST), as this latter provides a supraphysiological stimulus capable of increasing responsiveness of the partially damaged adrenal [112, 114]. On the other hand, diagnostic accuracy of the LDSST is affected by technical details related to the difficulty of preparing and delivering a very small amount of ACTH (1 µg through dilution of the initial 250 µg vial), with the consequent risk of administering inaccurate doses [115].

There is no clear evidence to indicate that LDSST is superior to SSST in the assessment of the HPA axis in children and both tests had similar diagnostic accuracy. Therefore, the choice of either SSST or LDSST should be individualized based on clinical judgment for each patient [113, 116].

In general, testing for CAI is still a clinical challenge because of the lack of standardized cortisol assays or evidence-based thresholds for diagnosis [117, 118].

Traditionally, peak cortisol concentrations > 18 µg/dl (500 nmol/L) after SDSST suggest a normal adrenal reserve [110] while for the LDSST values greater than 22 µg/dl (600 nmol/liter) best predicted normal adrenal function [9, 112, 119].

However, because these thresholds are based on older serum assays having high cross reactivity with non- cortisol steroids and the response to stimulation is always method specific, checking the reference range of the laboratory is recommended [1, 7].

Indeed, when interpreting both morning or stimulated cortisol, several issues should be considered. First, clinicians must be aware of the assay used in their local laboratory. The polyclonal antibody immunoassay has relatively poor specificity due to varying degrees of antibody cross-reactivity with endogenous proteins. In a clinical setting, this can potentially lead to overdiagnosis of AI and unnecessary use of replacement steroids. In many institutions, this assay has been recently replaced by monoclonal antibody (mAb) immunoassay or by liquid chromatography tandem mass spectrometry (LC/MS) [120], which have been demonstrated to be more specific. With the introduction of these newer assays, a redefinition of the diagnostic cortisol cut-offs appeared appropriate and a cortisol cut-off after SDSST of 14 to 15 µg/dl depending on the assay has been recently suggested to confirm or exclude adrenal insufficiency [120].

Second, factors like timing of cortisol sampling relative to ACTH administration (30–60 min after the injection), medications affecting cortisol binding and time of the day may interfere with results [119, 121, 122].

Finally, recent studies in adults indicate a promising role for home waking salivary cortisone (which reflects free serum cortisol) analyzed by using LC-MS/MS to overcome current limitations in the diagnosis of AI [123].

Other tests less commonly used in pediatric practice are represented by the insulin tolerance test (ITT) and overnight metyrapone stimulation test. ITT assesses the cortisol response to insulin-induced hypoglycemia and is considered the gold standard for the evaluation of HPA axis function. However, it is rarely used in children for the risk of severe hypoglycemia and is contraindicated in children with a history of seizures or cardiac disorders [9, 122].

Metyrapone inhibits the CYP11B1 enzyme that converts 11-deoxycortisol into active cortisol. Its administration results in a decrease of the negative feedback normally induced by cortisol on the HPA axis, followed by a surge of CRH and ACTH stimulating adrenal steroidogenesis. The inability of the HPA axis to respond appropriately is demonstrated by lower-than-normal increases in 11-deoxycortisol, the immediate precursor of cortisol. However, the metyrapone test is not recommended in children, due to the significant risk of acute adrenal crisis [122].

Dynamic testing should be the initial approach in situations where the assessment of morning cortisol lacks specificity. This is the case of infants or children with immaturity of the cortisol circadian rhythm and/or altered sleep – wake cycle that can be exacerbated by stressful factors, such as prolonged hospitalization and/or treatments and/or invasive procedures [124] (Fig. 2). Furthermore, it is worth noting that normal morning cortisol concentrations in children chronically exposed to GCs might not guarantee a proper cortisol increase during stressful events and thus do not reliably rule out AI [73]. Hence, in symptomatic patients a low threshold of suspicion for GI-AI should be kept even if basal cortisol is normal, suggesting the need for further testing.

## Treatment of GI-AI and educational needs

In children experiencing symptoms of AI or a relapse of the underlying disease during steroid tapering, GC treatment should be re-established, until recovery of the HPA axis is demonstrated [9, 59] (Fig. 2). In children who eventually discontinue GCs, full recovery of the HPA axis may occur after more than one year [61, 68].

Children with confirmed AI should receive daily hydrocortisone replacement treatment and then be periodically retested (Fig. 2).

In children with blunted cortisol response, it is still controversial whether daily GC replacement is necessary or a “stress dose only” approach should be considered [11, 47]. Indeed, many subjects with documented GI-AI may be asymptomatic, as the adrenal cortex might be able to produce steroids in sufficient quantities to satisfy normal daily needs, while under stressful conditions the adrenal cortex may not be able to increase cortisol production to meet extra requirements, leading to AI symptoms [5, 125] (Fig. 2). Therefore, it is suggested to consider treatment on a case-by-case basis, based on age (preferable to start in preverbal children), risk of relapse of the underlying condition, reliability and/or will of the parents.

Thus, educating patients and their families on the critical importance of initiating GC replacement therapy at the onset of AI symptoms, during intercurrent illnesses, pain, physical trauma, strenuous exercise or before stressful procedures or procedures requiring sedation is essential in managing GI-AI. It is of equal importance to adjust the dosage on sick days or prior to surgical interventions to prevent AC in those patients on daily GC replacement [5, 9, 125].

Therapeutic management of GI-AI is summarized in Table 5. As for other forms of AI, hydrocortisone remains the glucocorticoid of choice for GI-AI, as it allows a more physiological substitution [5]. It should be given at a dose of about 8 mg/m2/day in two to three doses, with the highest dose given in the morning [5] (Table 5). Given the lack of good quality evidence regarding the optimal dosing, current recommendations are based on expert consensus and/or local practice [125]. Although three daily doses of hydrocortisone allow a more physiological replacement therapy, it has been postulated that two daily doses, with avoidance of late afternoon dose, might favor recovery of the HPA axis, along with preventing symptoms of AI [4]. Doubling or tripling the oral dose of hydrocortisone is generally needed for the entire duration of a moderate febrile illness or infections severe enough to prevent the child from going to school [5, 9, 125]. Increasing the dose by one or two doses at most may also be considered for vaccinations [125] (Table 5). If possible, the patients should be provided with emergency contacts of the local pediatrician or pediatric endocrinology team, allowing them to seek advice regarding further management of the intercurrent illness [5, 9, 125]. When the patient is unable to take steroids orally, for example due to vomiting, drowsiness, unconsciousness, or major trauma, hydrocortisone must be given intramuscularly before seeking emergency care or visiting the nearest emergency department [5, 125] (Table 5).

**Table 5:** Therapeutic management of glucocorticoid-induced adrenal insufficiency

	Hydrocortisone dose regimen	Additional measures	Follow-up
Standard replacement	8–10 mg/m^2^/day in 2 or 3 doses with the highest in the morning	Education of family and healthcare providers regarding management of AI and AC Emergency steroid card/bracelet/necklace Hydrocortisone emergency self-injection kit	Reassess the HPA axis every 1–3 months until recovery is documented (Fig. 2).
Minor febrile illness	Double the usual dose.	As for glucocorticoid replacement If oral administration is not tolerated (es. vomiting or diarrhea) use im injection	Clinical monitoring Contact medical team if not improving within 1–2 days Go back to the usual dose when clinically improved.
Vaccinations	Consider doubling 1 or 2 doses		
Adrenal crisis	Initial iv or im bolus - less than 1 year: 25 mg* - 1–5 year: 50 mg - 6 years and over: 100 mg followed by hydrocortisone 50 to 75 mg/m2/day divided in 6-hourly iv/im boluses or as continuous iv infusion	Contact emergency services (possibly after initial bolus) and arrange hospital admission for further management Ensure glucose and electrolytes homeostasis with maintenance glucosaline infusion Treat hypoglycemia with 2 ml/kg of 10% dextrose iv bolus and repeat if still hypoglycemic after 15 min Treat hypovolemic shock with 10 ml/kg boluses of saline solution 0.9%.	When clinically settled and oral intake tolerated, switch to oral hydrocortisone 30 mg/m2/day in four divided doses and then reduce to maintenance dose gradually in 2–3 days.
Minor surgery or procedures under local anesthetic or sedation	Double or triple steroid dose before and after the procedure	Inform doctors regarding AI and provide steroidal card revised by the endocrine team in charge.	Keep double or triple dose of hydrocortisone up to 24 h if prolonged pain or malaise.

*Abbreviations* HPA, hypothalamic-pituitary-adrenal; im, intramuscular; iv, intravenous

* In neonates (< 28 days corrected chronological age) iv hydrocortisone bolus 4 mg/kg

# In neonates (< 28 days corrected chronological age) iv hydrocortisone bolus 2 mg/kg

Regardless of whether treatment has been started or not, the patient must be supplied with hydrocortisone injections, and trained to perform them in case of suspected or overt AC, before seeking medical attention [5, 125] (Table 5). All individuals with adrenal insufficiency (AI) and their parents must be advised to inform their healthcare providers about their AI diagnosis before undergoing any urgent or elective medical (e.g., endoscopy) or surgical procedures [5]. Moreover, they should carry an emergency bracelet or necklace or a steroid card reporting details of the underlying condition, the current treatment regimen and emergency treatment [5, 125, 126]. These identification tools must be periodically reviewed with the family and customized according to the patient’s anthropometry and/or specific needs [5, 125, 126].

Abrupt withdrawal of GC treatment, quick tapering or stressful events in patients with treated or untreated GI-AI can precipitate an acute adrenal crisis. This is characterized by fainting and/or circulatory collapse and/or seizures without suspicion of any other underlying disease, even though subtle forms may present with anorexia, fatigue, nausea, vomiting, dyspnea, myalgia, and orthostatic hypotension [127] (Table 2). Thus, education of health care providers regarding recognition and management of adrenal crisis is necessary. To enhance safety and improve treatment outcomes for patients during hospital admissions, especially in emergency situations when they do not have their medical documentation, integrating medical alerts into electronic health records may be a key strategy [128]. These alerts should indicate whether the patient has AI or is undergoing chronic GC treatment, functioning like an emergency card [128].

## Open issues/future perspectives

GCs represent the mainstay of treatment for several acute and chronic pediatric diseases. Although it is well known that withdrawal of GCs after prolonged treatment may be associated with prolonged AI, this is still the cause of significant morbidity and mortality. The underlying reasons include widespread underestimation of the effects of different steroid treatments on the HPA axis in children.

A universally valid tapering protocol is probably not reasonably conceivable, because of the peculiar clinical behavior of each underlying disease. Nevertheless, randomized controlled studies comparing efficacy and safety of different GC tapering protocols in specific pediatric diseases are currently lacking, resulting in very heterogeneous management.

At present, one of the most critical points is the inability to accurately estimate the risk of GI-AI, partly due to the presence of constitutional and/or genetic factors. Hence, in the era of precision medicine it is desirable that further efforts are made to identify individual markers predicting response to GCs and/or recovery rate of the HPA axis, thus allowing a tailored approach to the patient.

We have emphasized the importance of screening for GI-AI subjects at higher risk of developing such condition. To date, too many cases are still diagnosed only when they become symptomatic or during acute hospital admission due to AC. However, specificity of the diagnostic tests available for diagnosis of CAI needs to be optimized, and new reference ranges are required when using news assays and methods in children.

Even in subjects who do not need to be tested, prompt recognition of symptoms of GI-AI is of paramount importance to allow timely treatment and prevention of AC. Therefore, a key point is education of both families and health care providers regarding the pitfalls of GC withdrawal and management of AI. For this purpose, wide implementation of medical alerts on electronic healthcare records or other similar strategies should be encouraged.

## Data Availability

Data sharing not applicable to this article as no datasets were generated or analysed during the current study.

## References

[1] van Staa TP, Leufkens HG, Abenhaim L, Begaud B, Zhang B, Cooper C (2000) Use of oral corticosteroids in the United Kingdom. QJM 93(2):105–111. 10.1093/qjmed/93.2.105 10700481 10.1093/qjmed/93.2.105

[2] Bénard-Laribière A, Pariente A, Pambrun E, Bégaud B, Fardet L, Noize P (2017) Prevalence and prescription patterns of oral glucocorticoids in adults: a retrospective cross-sectional and cohort analysis in France. BMJ Open 7(7):e015905. 10.1136/bmjopen-2017-015905 28760791 10.1136/bmjopen-2017-015905 PMC5642779

[3] Broersen LH, Pereira AM, Jørgensen JO, Dekkers OM (2015) Adrenal insufficiency in corticosteroids Use: systematic review and Meta-analysis. J Clin Endocrinol Metab 100(6):2171–2180. 10.1210/jc.2015-1218 25844620 10.1210/jc.2015-1218

[4] Prete A, Bancos I (2021) Glucocorticoid induced adrenal insufficiency. BMJ 374:n1380. 10.1136/bmj.n1380. Erratum in: BMJ. 2021;374:n1936 10.1136/bmj.n1380 34253540

[5] Charmandari E, Nicolaides NC, Chrousos GP (2014) Adrenal insufficiency. Lancet 383(9935):2152–2167. 10.1016/S0140-6736(13)61684-0 24503135 10.1016/S0140-6736(13)61684-0

[6] Dinsen S, Baslund B, Klose M, Rasmussen AK, Friis-Hansen L, Hilsted L, Feldt-Rasmussen U (2013) Why glucocorticoid withdrawal may sometimes be as dangerous as the treatment itself. Eur J Intern Med 24(8):714–720. 10.1016/j.ejim.2013.05.014. Erratum in: Eur J Intern Med. 2014;25(8):781-3 10.1016/j.ejim.2013.05.014 23806261

[7] Capalbo D, Moracas C, Cappa M et al (2021) Primary adrenal insufficiency in Childhood: Data from a large Nationwide Cohort. J Clin Endocrinol Metab 106(3):762–773. 10.1210/clinem/dgaa881 33247909 10.1210/clinem/dgaa881

[8] Hahner S, Ross RJ, Arlt W, Bancos I, Burger-Stritt S, Torpy DJ, Husebye ES, Quinkler M (2021) Adrenal insufficiency. Nat Rev Dis Primers 7(1):19. 10.1038/s41572-021-00252-7 33707469 10.1038/s41572-021-00252-7

[9] Patti G, Guzzetti C, Di Iorgi N, Maria Allegri AE, Napoli F, Loche S, Maghnie M (2018) Central adrenal insufficiency in children and adolescents. Best Pract Res Clin Endocrinol Metab 32(4):425–444. 10.1016/j.beem.2018.03.012 30086867 10.1016/j.beem.2018.03.012

[10] Ahmet A, Mokashi A, Goldbloom EB, Huot C, Jurencak R, Krishnamoorthy P, Rowan-Legg A, Kim H, Pancer L, Kovesi T (2019) Adrenal suppression from glucocorticoids: preventing an iatrogenic cause of morbidity and mortality in children. BMJ Paediatr Open 3(1):e000569. 10.1136/bmjpo-2019-000569 31750407 10.1136/bmjpo-2019-000569 PMC6830460

[11] Borresen SW, Klose M, Glintborg D, Watt T, Andersen MS, Feldt-Rasmussen U (2022) Approach to the patient with glucocorticoid-induced adrenal insufficiency. J Clin Endocrinol Metab 107(7):2065–2076. 10.1210/clinem/dgac151 35302603 10.1210/clinem/dgac151

[12] Arlt W, Allolio B (2003) Adrenal insufficiency. Lancet 361(9372):1881–1893. 10.1016/S0140-6736(03)13492-7 12788587 10.1016/S0140-6736(03)13492-7

[13] Goldbloom EB, Mokashi A, Cummings EA, Abish S, Benseler SM, Huynh HQ, Watson W, Ahmet A (2017) Symptomatic adrenal suppression among children in Canada. Arch Dis Child 102(4):338–339. 10.1136/archdischild-2016-311223 28320817 10.1136/archdischild-2016-311223

[14] Joseph RM, Hunter AL, Ray DW, Dixon WG (2016) Systemic glucocorticoid therapy and adrenal insufficiency in adults: a systematic review. Semin Arthritis Rheum 46(1):133–141. 10.1016/j.semarthrit.2016.03.001 27105755 10.1016/j.semarthrit.2016.03.001 PMC4987145

[15] Wildi-Runge S, Deladoëy J, Bélanger C, Deal CL, Van Vliet G, Alos N, Huot C (2013) A search for variables predicting cortisol response to low-dose corticotropin stimulation following supraphysiological doses of glucocorticoids. J Pediatr 163(2):484–488. 10.1016/j.jpeds.2013.01.011 23414662 10.1016/j.jpeds.2013.01.011

[16] Laulhé M, Dumaine C, Chevenne D et al (2022) Glucocorticoid induced adrenal insufficiency in children: morning cortisol values to avoid LDSST. Front Pediatr 10:981765. 10.3389/fped.2022.981765 36589156 10.3389/fped.2022.981765 PMC9798323

[17] Sidoroff M, Kolho KL (2014) Screening for adrenal suppression in children with inflammatory bowel disease discontinuing glucocorticoid therapy. BMC Gastroenterol 14:51. 10.1186/1471-230X-14-51 24661924 10.1186/1471-230X-14-51 PMC3987131

[18] Krasner AS (1999) Glucocorticoid-induced adrenal insufficiency. JAMA 282(7):671–676. 10.1001/jama.282.7.671 10517721 10.1001/jama.282.7.671

[19] Bancos I, Hahner S, Tomlinson J, Arlt W (2015) Diagnosis and management of adrenal insufficiency. Lancet Diabetes Endocrinol 3(3):216–226. 10.1016/S2213-8587(14)70142-1 25098712 10.1016/S2213-8587(14)70142-1

[20] Bizzarri C, Capalbo D, Wasniewska MG, Baronio F, Grandone A, Cappa M (2023) Adrenal crisis in infants and young children with adrenal insufficiency: management and prevention. Front Endocrinol (Lausanne) 14:1133376. 10.3389/fendo.2023.1133376 36860362 10.3389/fendo.2023.1133376 PMC9968740

[21] Eyal O, Levin Y, Oren A et al (2019) Adrenal crises in children with adrenal insufficiency: epidemiology and risk factors. Eur J Pediatr 178(5):731–738. 10.1007/s00431-019-03348-1 30806790 10.1007/s00431-019-03348-1

[22] Li D, Genere N, Behnken E, Xhikola M, Abbondanza T, Vaidya A, Bancos I (2021) Determinants of self-reported Health outcomes in adrenal insufficiency: a Multisite Survey Study. J Clin Endocrinol Metab 106(3):e1408–e1419. 10.1210/clinem/dgaa668 32995875 10.1210/clinem/dgaa668 PMC7947833

[23] Rushworth RL, Torpy DJ, Falhammar H (2019) Adrenal Crisis. N Engl J Med 381(9):852–861. 10.1056/NEJMra1807486 31461595 10.1056/NEJMra1807486

[24] Esteban NV, Loughlin T, Yergey AL, Zawadzki JK, Booth JD, Winterer JC, Loriaux DL (1991) Daily cortisol production rate in man determined by stable isotope dilution/mass spectrometry. J Clin Endocrinol Metab 72(1):39–45. 10.1210/jcem-72-1-39 1986026 10.1210/jcem-72-1-39

[25] Peters CJ, Hill N, Dattani MT et al (2013) Deconvolution analysis of 24-h serum cortisol profiles informs the amount and distribution of hydrocortisone replacement therapy. Clin Endocrinol (Oxf) 78:347–351. 10.1111/j.1365-2265.2012.04502.x 22803584 10.1111/j.1365-2265.2012.04502.x

[26] Auer MK, Nordenström A, Lajic S, Reisch N (2023) Congenital adrenal hyperplasia. Lancet 401(10372):227–244. 10.1016/S0140-6736(22)01330-7 36502822 10.1016/S0140-6736(22)01330-7

[27] Lamberts SW, Bruining HA, de Jong FH (1997) Corticosteroid therapy in severe illness. N Engl J Med 337(18):1285–1292. 10.1056/NEJM199710303371807 9345079 10.1056/NEJM199710303371807

[28] Cooper MS, Stewart PM (2003) Corticosteroid insufficiency in acutely ill patients. N Engl J Med 348(8):727–734. 10.1056/NEJMra020529 12594318 10.1056/NEJMra020529

[29] Improda N, Capalbo D, Poloniato A et al (2023) Perinatal asphyxia and hypothermic treatment from the endocrine perspective. Front Endocrinol (Lausanne) 14:1249700. 10.3389/fendo.2023.1249700 37929024 10.3389/fendo.2023.1249700 PMC10623321

[30] Nicolaides NC, Kino T, Roberts ML, Katsantoni E, Sertedaki A, Moutsatsou P, Psarra AG, Chrousos GP, Charmandari E (2017) The role of S-Palmitoylation of the human glucocorticoid receptor (hGR) in mediating the nongenomic glucocorticoid actions. J Mol Biochem 6(1):3–12 28775968 PMC5538142

[31] Rovin BH, Adler SG, Barratt J et al (2021) Executive summary of the KDIGO 2021 Guideline for the management of glomerular diseases. Kidney Int 100(4):753–779. 10.1016/j.kint.2021.05.015 34556300 10.1016/j.kint.2021.05.015

[32] Okamoto N, Yokota S, Takei S et al (2019) Clinical practice guidance for juvenile idiopathic arthritis (JIA) 2018. Mod Rheumatol 29(1):41–59. 10.1080/14397595.2018.1514724 30126298 10.1080/14397595.2018.1514724

[33] Ruemmele FM, Veres G, Kolho KL et al (2014) Consensus guidelines of ECCO/ESPGHAN on the medical management of pediatric Crohn’s disease. J Crohns Colitis 8(10):1179–1207. 10.1016/j.crohns.2014.04.005 24909831 10.1016/j.crohns.2014.04.005

[34] Turner D, Ruemmele FM, Orlanski-Meyer E et al (2018) Management of Paediatric Ulcerative Colitis, Part 1: Ambulatory Care-An evidence-based Guideline from European Crohn’s and Colitis Organization and European Society of Paediatric Gastroenterology, Hepatology and Nutrition. J Pediatr Gastroenterol Nutr 67(2):257–291. 10.1097/MPG.0000000000002035. Erratum in: J Pediatr Gastroenterol Nutr. 2020;71(6):794 30044357 10.1097/MPG.0000000000002035

[35] Mieli-Vergani G, Vergani D, Baumann U et al (2018) Diagnosis and management of Pediatric Autoimmune Liver Disease: ESPGHAN Hepatology Committee position Statement. J Pediatr Gastroenterol Nutr 66(2):345–360. 10.1097/MPG.0000000000001801 29356770 10.1097/MPG.0000000000001801

[36] Olivier-Gougenheim L, Arfeuille C, Suciu S et al (2020) Pediatric randomized trial EORTC CLG 58951: outcome for adolescent population with acute lymphoblastic leukemia. Hematol Oncol 38(5):763–772. 10.1002/hon.2791 32809224 10.1002/hon.2791

[37] Weissinger EM, Metzger J, Schleuning M et al (2021) A multicenter prospective, randomized, placebo-controlled phase II/III trial for preemptive acute graft-versus-host disease therapy. Leukemia 35(6):1763–1772. 10.1038/s41375-020-01059-3 33082512 10.1038/s41375-020-01059-3 PMC8179847

[38] Ackerman GL, Nolsn CM (1968) Adrenocortical responsiveness after alternate-day corticosteroid therapy. N Engl J Med 278(8):405–409. 10.1056/NEJM196802222780801 5636662 10.1056/NEJM196802222780801

[39] Bleecker ER, Menzies-Gow AN, Price DB, Bourdin A, Sweet S, Martin AL, Alacqua M, Tran TN (2020) Systematic literature review of systemic corticosteroid use for Asthma Management. Am J Respir Crit Care Med 201(3):276–293. 10.1164/rccm.201904-0903SO 31525297 10.1164/rccm.201904-0903SO PMC6999108

[40] Gupta R, Fonacier LS (2016) Adverse effects of nonsystemic steroids (inhaled, Intranasal, and cutaneous): a review of the literature and suggested Monitoring Tool. Curr Allergy Asthma Rep 16(6):44. 10.1007/s11882-016-0620-y 27207481 10.1007/s11882-016-0620-y

[41] Yu SH, Drucker AM, Lebwohl M, Silverberg JI (2018) A systematic review of the safety and efficacy of systemic corticosteroids in atopic dermatitis. J Am Acad Dermatol 78(4):733–740e11. 10.1016/j.jaad.2017.09.074 29032119 10.1016/j.jaad.2017.09.074

[42] Liu D, Ahmet A, Ward L, Krishnamoorthy P, Mandelcorn ED, Leigh R, Brown JP, Cohen A, Kim H (2013) A practical guide to the monitoring and management of the complications of systemic corticosteroid therapy. Allergy Asthma Clin Immunol 9(1):30. 10.1186/1710-1492-9-30 23947590 10.1186/1710-1492-9-30 PMC3765115

[43] Shulman DI, Palmert MR, Kemp SF, Lawson Wilkins Drug and Therapeutics Committee (2007) Adrenal insufficiency: still a cause of morbidity and death in childhood. Pediatrics 119(2):e484–494. 10.1542/peds.2006-1612 17242136 10.1542/peds.2006-1612

[44] Lodish MB, Keil MF, Stratakis CA (2018) Cushing’s syndrome in Pediatrics: an update. Endocrinol Metab Clin North Am 47(2):451–462. 10.1016/j.ecl.2018.02.008 29754644 10.1016/j.ecl.2018.02.008 PMC5962291

[45] Ciccone S, Marini R, Bizzarri C, El Hachem M, Cappa M (2016) Cushing’s syndrome in a 6-month-old boy: a rare side-effect due to inadequate use of topical corticosteroids. Acta Derm Venereol 96(1):138–139. 10.2340/00015555-2151 26038976 10.2340/00015555-2151

[46] Borresen SW, Klose M, Rasmussen AK, Feldt-Rasmussen U (2015) Adrenal insufficiency caused by locally Applied glucocorticoids-myth or fact? Curr Med Chem 22(23):2801–2809. 10.2174/0929867322666150716113003 26180005 10.2174/0929867322666150716113003

[47] Perry RJ, Findlay CA, Donaldson MD (2002) Cushing’s syndrome, growth impairment, and occult adrenal suppression associated with intranasal steroids. Arch Dis Child 87(1):45–48. 10.1136/adc.87.1.45 12089123 10.1136/adc.87.1.45 PMC1751129

[48] Pofi R, Caratti G, Ray DW, Tomlinson JW (2003) Treating the Side effects of Exogenous glucocorticoids; can we separate the good from the bad? Endocr Rev 44(6):975–1011. 10.1210/endrev/bnad016 10.1210/endrev/bnad016 PMC10638606 37253115

[49] Ahmet A, Kim H, Spier S (2011) Adrenal suppression: a practical guide to the screening and management of this under-recognized complication of inhaled corticosteroid therapy. Allergy Asthma Clin Immunol 7(1):13. 10.1186/1710-1492-7-13 21867553 10.1186/1710-1492-7-13 PMC3177893

[50] Hengge UR, Ruzicka T, Schwartz RA, Cork MJ (2006) Adverse effects of topical glucocorticosteroids. J Am Acad Dermatol 54(1):1–15. 10.1016/j.jaad.2005.01.010 16384751 10.1016/j.jaad.2005.01.010

[51] Carruthers JA, August PJ, Staughton RC (1975) Observations on the systemic effect of topical clobetasol propionate (Dermovate). Br Med J 4(5990):203–204. 10.1136/bmj.4.5990.203 1191997 10.1136/bmj.4.5990.203 PMC1674965

[52] Molimard M, Girodet PO, Pollet C, Fourrier-Réglat A, Daveluy A, Haramburu F, Fayon M, Tabarin A (2008) Inhaled corticosteroids and adrenal insufficiency: prevalence and clinical presentation. Drug Saf 31(9):769–774. 10.2165/00002018-200831090-00005 18707191 10.2165/00002018-200831090-00005

[53] Paton J, Jardine E, McNeill E, Beaton S, Galloway P, Young D, Donaldson M (2006) Adrenal responses to low dose synthetic ACTH (Synacthen) in children receiving high dose inhaled fluticasone. Arch Dis Child 91(10):808–813. 10.1136/adc.2005.087247 16556614 10.1136/adc.2005.087247 PMC2066000

[54] Graber AL, Ney RL, Nicholson WE, Island DP, Liddle GW (1965) Natural history of pituitary-adrenal recovery following long-term suppression with corticosteroids. J Clin Endocrinol Metab 25:11–16. 10.1210/jcem-25-1-11 14252277 10.1210/jcem-25-1-11

[55] Bayman E, Drake AJ (2017) Adrenal suppression with glucocorticoid therapy: still a problem after all these years? Arch Dis Child 102(4):338–339. 10.1136/archdischild-2016-311601 27879240 10.1136/archdischild-2016-311601

[56] Einaudi S, Bertorello N, Masera N et al (2008) Adrenal axis function after high-dose steroid therapy for childhood acute lymphoblastic leukemia. Pediatr Blood Cancer 50(3):537–341. 10.1002/pbc.21339 17828747 10.1002/pbc.21339

[57] Rensen N, Gemke RJ, van Dalen EC, Rotteveel J, Kaspers GJ (2017) Hypothalamic-pituitary-adrenal (HPA) axis suppression after treatment with glucocorticoid therapy for childhood acute lymphoblastic leukaemia. Cochrane Database Syst Rev 11(11):CD008727. 10.1002/14651858.CD008727.pub4 29106702 10.1002/14651858.CD008727.pub4 PMC6486149

[58] Einarsdottir MJ, Trimpou P, Johannsson G, Ragnarsson O (2024) Undiagnosed adrenal insufficiency as a cause of premature death in glucocorticoid users. Endocr Connect 13(4):e230535. 10.1530/EC-23-0535 38428141 10.1530/EC-23-0535 PMC10959030

[59] Hochberg Z, Pacak K, Chrousos GP (2003) Endocrine withdrawal syndromes. Endocr Rev 24(4):523–538. 10.1210/er.2001-0014 12920153 10.1210/er.2001-0014

[60] Amatruda TT Jr, Hurst MM, D’Esopo ND (1965) Certain endocrine and metabolic facets of the steroid withdrawal syndrome. J Clin Endocrinol Metab 25(9):1207–1217. 10.1210/jcem-25-9-1207 4284084 10.1210/jcem-25-9-1207

[61] Hurtado MD, Cortes T, Natt N, Young WF Jr, Bancos I (2018) Extensive clinical experience: hypothalamic-pituitary-adrenal axis recovery after adrenalectomy for corticotropin-independent cortisol excess. Clin Endocrinol (Oxf) 89(6):721–733. 10.1111/cen.13803 29968420 10.1111/cen.13803 PMC6246804

[62] Saracco P, Bertorello N, Farinasso L, Einaudi S, Barisone E, Altare F, Corrias A, Pastore G (2005) Steroid withdrawal syndrome during steroid tapering in childhood acute lymphoblastic leukemia: a controlled study comparing prednisone versus dexamethasone in induction phase. J Pediatr Hematol Oncol 27(3):141–144. 10.1097/01.mph.0000155870.38794.e7. Erratum in: J Pediatr Hematol Oncol. 2005;27(4):242 15750445 10.1097/01.mph.0000155870.38794.e7

[63] Papanicolaou DA, Tsigos C, Oldfield EH, Chrousos GP (1996) Acute glucocorticoid deficiency is associated with plasma elevations of interleukin-6: does the latter participate in the symptomatology of the steroid withdrawal syndrome and adrenal insufficiency? J Clin Endocrinol Metab 81(6):2303–2306. 10.1210/jcem.81.6.8964868 8964868 10.1210/jcem.81.6.8964868

[64] Schlaghecke R, Kornely E, Santen RT, Ridderskamp P (1992) The effect of long-term glucocorticoid therapy on pituitary-adrenal responses to exogenous corticotropin-releasing hormone. N Engl J Med 326(4):226–230. 10.1056/NEJM199201233260403 1309389 10.1056/NEJM199201233260403

[65] Spiegel RJ, Vigersky RA, Oliff AI, Echelberger CK, Bruton J, Poplack DG (1979) Adrenal suppression after short-term corticosteroid therapy. Lancet 1(8117):630–633. 10.1016/s0140-6736(79)91077-8 85870 10.1016/s0140-6736(79)91077-8

[66] Dolan LM, Kesarwala HH, Holroyde JC, Fischer TJ (1987) Short-term, high-dose, systemic steroids in children with asthma: the effect on the hypothalamic-pituitary-adrenal axis. J Allergy Clin Immunol 80(1):81–87. 10.1016/s0091-6749(87)80195-1 3598031 10.1016/s0091-6749(87)80195-1

[67] Alves C, Robazzi TC, Mendonça M (2008) Withdrawal from glucocorticosteroid therapy: clinical practice recommendations. J Pediatr (Rio J) 84(3):192–202. 10.2223/JPED.1773 18535733 10.2223/JPED.1773

[68] Sampieri G, Namavarian A, Lee JJW, Hamour AF, Lee JM (2022) Hypothalamic-pituitary-adrenal axis suppression and intranasal corticosteroid use: a systematic review and meta-analysis. Int Forum Allergy Rhinol 12(1):11–27. 10.1002/alr.22863 34260153 10.1002/alr.22863

[69] Bruni FM, De Luca G, Venturoli V, Boner AL (2009) Intranasal corticosteroids and adrenal suppression. Neuroimmunomodulation 16(5):353–362. 10.1159/000216193 19571596 10.1159/000216193

[70] Zöllner EW, Lombard CJ, Galal U, Hough FS, Irusen EM, Weinberg E (2012) Hypothalamic-pituitary-adrenal axis suppression in asthmatic school children. Pediatrics 130(6):e1512–1519. 10.1542/peds.2012-1147 23147980 10.1542/peds.2012-1147

[71] Zöllner EW, Lombard CJ, Galal U, Hough S, Irusen EM, Weinberg E (2013) Screening for hypothalamic-pituitary-adrenal axis suppression in asthmatic children remains problematic: a cross-sectional study. BMJ Open 3(8):e002935. 10.1136/bmjopen-2013-002935 23906954 10.1136/bmjopen-2013-002935 PMC3733311

[72] Wood Heickman LK, Davallow Ghajar L, Conaway M, Rogol AD (2018) Evaluation of hypothalamic-pituitary-adrenal Axis suppression following cutaneous use of topical corticosteroids in children: a Meta-analysis. Horm Res Paediatr 89(6):389–396. 10.1159/000489125 29898449 10.1159/000489125

[73] Kapadia CR, Nebesio TD, Myers SE, Willi S, Miller BS, Allen DB, Jacobson-Dickman E, Drugs, Therapeutics Committee of the Pediatric Endocrine Society (2016) Endocrine effects of Inhaled corticosteroids in Children. JAMA Pediatr 170(2):163–170. 10.1001/jamapediatrics.2015.3526 26720105 10.1001/jamapediatrics.2015.3526

[74] Allen DB (2020) Inhaled corticosteroids and Endocrine effects in Childhood. Endocrinol Metab Clin North Am 49(4):651–665. 10.1016/j.ecl.2020.07.003 33153672 10.1016/j.ecl.2020.07.003

[75] Busse WW, Brazinsky S, Jacobson K et al (1999) Efficacy response of inhaled beclomethasone dipropionate in asthma is proportional to dose and is improved by formulation with a new propellant. J Allergy Clin Immunol 104(6):1215–1222. 10.1016/s0091-6749(99)70016-3 10589004 10.1016/s0091-6749(99)70016-3

[76] Harrison LI, Colice GL, Donnell D et al (1999) Adrenal effects and pharmacokinetics of CFC-free beclomethasone dipropionate: a 14-day dose-response study. J Pharm Pharmacol 51(3):263–269. 10.1211/0022357991772439 10344626 10.1211/0022357991772439

[77] Martinez FD, Chinchilli VM, Morgan WJ et al (2011) Use of beclomethasone dipropionate as rescue treatment for children with mild persistent asthma (TREXA): a randomised, double-blind, placebo-controlled trial. Lancet 377(9766):650–657. 10.1016/S0140-6736(10)62145-9 21324520 10.1016/S0140-6736(10)62145-9 PMC4852146

[78] Issa-El-Khoury K, Kim H, Chan ES, Vander Leek T, Noya F (2015) CSACI position statement: systemic effect of inhaled corticosteroids on adrenal suppression in the management of pediatric asthma. Allergy Asthma Clin Immunol 11(1):9. 10.1186/s13223-015-0075-z 25802532 10.1186/s13223-015-0075-z PMC4369840

[79] Rao Bondugulapati LN, Rees DA (2016) Inhaled corticosteroids and HPA axis suppression: how important is it and how should it be managed? Clin Endocrinol (Oxf) 85(2):165–169. 10.1111/cen.13073 27038017 10.1111/cen.13073

[80] Allen A, Bareille PJ, Rousell VM (2013) Fluticasone furoate, a novel inhaled corticosteroid, demonstrates prolonged lung absorption kinetics in man compared with inhaled fluticasone propionate. Clin Pharmacokinet 52(1):37–42. 10.1007/s40262-012-0021-x 23184737 10.1007/s40262-012-0021-x PMC3693428

[81] Allen A, Bal J, Cheesbrough A, Hamilton M, Kempsford R (2014) Pharmacokinetics and pharmacodynamics of intravenous and inhaled fluticasone furoate in healthy caucasian and east Asian subjects. Br J Clin Pharmacol 77(5):808–820. 10.1111/bcp.12263 24152086 10.1111/bcp.12263 PMC4004401

[82] Golekoh MC, Hornung LN, Mukkada VA, Khoury JC, Putnam PE, Backeljauw PF (2016) Adrenal insufficiency after chronic swallowed glucocorticoid therapy for eosinophilic esophagitis. J Pediatr 170:240–245. 10.1016/j.jpeds.2015.11.026 26687577 10.1016/j.jpeds.2015.11.026

[83] Philla KQ, Min SB, Hefner JN, Howard RS, Reinhardt BJ, Nazareno LG, Vogt KS (2015) Swallowed glucocorticoid therapy for eosinophilic esophagitis in children does not suppress adrenal function. J Pediatr Endocrinol Metab 28(9–10):1101–1106. 10.1515/jpem-2014-0260 26024243 10.1515/jpem-2014-0260

[84] Ahmet A, Benchimol EI, Goldbloom EB, Barkey JL (2016) Adrenal suppression in children treated with swallowed fluticasone and oral viscous budesonide for eosinophilic esophagitis. Allergy Asthma Clin Immunol 12:49. 10.1186/s13223-016-0154-9 27766109 10.1186/s13223-016-0154-9 PMC5057375

[85] Cohen SA, Aloi M, Arumugam R, Baker R, Bax K, Kierkuś J, Koletzko S, Lionetti P, Persson T, Eklund S (2017) Enteric-coated budesonide for the induction and maintenance of remission of Crohn’s disease in children. Curr Med Res Opin 33(7):1261–1268. 10.1080/03007995.2017.1313213 28420280 10.1080/03007995.2017.1313213

[86] Seow CH, Benchimol EI, Griffiths AM, Otley AR, Steinhart AH (2008) Budesonide for induction of remission in Crohn’s disease. Cochrane Database Syst Rev 3:CD000296. 10.1002/14651858.CD000296.pub3. Update in: Cochrane Database Syst Rev (2015) 6:CD000296 10.1002/14651858.CD000296.pub4 PMC10613338 26039678

[87] Gionchetti P, D’Arienzo A, Rizzello F et al (2005) Topical treatment of distal active ulcerative colitis with beclomethasone dipropionate or mesalamine: a single-blind randomized controlled trial. J Clin Gastroenterol 39(4):291–297. 10.1097/01.mcg.0000155124.74548.61 15758622 10.1097/01.mcg.0000155124.74548.61

[88] Bonovas S, Nikolopoulos GK, Lytras T, Fiorino G, Peyrin-Biroulet L, Danese S (2018) Comparative safety of systemic and low-bioavailability steroids in inflammatory bowel disease: systematic review and network meta-analysis. Br J Clin Pharmacol 84(2):239–251. 10.1111/bcp.13456 29057539 10.1111/bcp.13456 PMC5777428

[89] Dort K, Padia S, Wispelwey B, Moore CC (2009) Adrenal suppression due to an interaction between ritonavir and injected triamcinolone: a case report. AIDS Res Ther 6:10. 10.1186/1742-6405-6-10 19505306 10.1186/1742-6405-6-10 PMC2701432

[90] Yombi JC, Maiter D, Belkhir L, Nzeusseu A, Vandercam B (2008) Iatrogenic cushing’s syndrome and secondary adrenal insufficiency after a single intra-articular administration of triamcinolone acetonide in HIV-infected patients treated with ritonavir. Clin Rheumatol 27(Suppl 2):S79–82. 10.1007/s10067-008-1022-x 18827959 10.1007/s10067-008-1022-x

[91] Saberi P, Phengrasamy T, Nguyen DP (2013) Inhaled corticosteroid use in HIV-positive individuals taking protease inhibitors: a review of pharmacokinetics, case reports and clinical management. HIV Med 14(9):519–529. 10.1111/hiv.12039 23590676 10.1111/hiv.12039 PMC3758391

[92] Dineen R, Stewart PM, Sherlock M (2019) Factors impacting on the action of glucocorticoids in patients receiving glucocorticoid therapy. Clin Endocrinol (Oxf) 90(1):3–14. 10.1111/cen.13837 30120786 10.1111/cen.13837

[93] Ekman B, Bachrach-Lindström M, Lindström T, Wahlberg J, Blomgren J, Arnqvist HJ (2012) A randomized, double-blind, crossover study comparing two- and four-dose hydrocortisone regimen with regard to quality of life, cortisol and ACTH profiles in patients with primary adrenal insufficiency. Clin Endocrinol (Oxf) 77(1):18–25. 10.1111/j.1365-2265.2012.04352.x 22288685 10.1111/j.1365-2265.2012.04352.x

[94] Charmandari E, Chrousos GP, Lambrou GI, Pavlaki A, Koide H, Ng SS, Kino T (2011) Peripheral CLOCK regulates target-tissue glucocorticoid receptor transcriptional activity in a circadian fashion in man. PLoS ONE 6(9):e25612. 10.1371/journal.pone.0025612 21980503 10.1371/journal.pone.0025612 PMC3182238

[95] Quax RA, Manenschijn L, Koper JW, Hazes JM, Lamberts SW, van Rossum EF, Feelders RA (2013) Glucocorticoid sensitivity in health and disease. Nat Rev Endocrinol 9(11):670–686. 10.1038/nrendo.2013.183 24080732 10.1038/nrendo.2013.183

[96] de Ruiter RD, Gordijn MS, Gemke RJ, van den Bos C, Bierings MB, Rotteveel J, Koper JW, van Rossum EF, Kaspers GL (2014) Adrenal insufficiency during treatment for childhood acute lymphoblastic leukemia is associated with glucocorticoid receptor polymorphisms ER22/23EK and BclI. Haematologica 99(8):e136–137. 10.3324/haematol.2014.105056 24816241 10.3324/haematol.2014.105056 PMC4116845

[97] Hawcutt DB, Francis B, Carr DF et al (2018) Susceptibility to corticosteroid-induced adrenal suppression: a genome-wide association study. Lancet Respir Med 6(6):442–450. 10.1016/S2213-2600(18)30058-4 29551627 10.1016/S2213-2600(18)30058-4 PMC5971210

[98] Derijk RH (2009) Single nucleotide polymorphisms related to HPA axis reactivity. Neuroimmunomodulation 16(5):340–352. 10.1159/000216192 19571595 10.1159/000216192

[99] Lou QY, Li Z, Teng Y et al (2021) Associations of FKBP4 and FKBP5 gene polymorphisms with disease susceptibility, glucocorticoid efficacy, anxiety, depression, and health-related quality of life in systemic lupus erythematosus patients. Clin Rheumatol 40(1):167–179. 10.1007/s10067-020-05195-0 32557257 10.1007/s10067-020-05195-0

[100] Lashansky G, Saenger P, Fishman K, Gautier T, Mayes D, Berg G, Di Martino-Nardi J, Reiter E (1991) Normative data for adrenal steroidogenesis in a healthy pediatric population: age- and sex-related changes after adrenocorticotropin stimulation. J Clin Endocrinol Metab 73(3):674–686. 10.1210/jcem-73-3-674 1651957 10.1210/jcem-73-3-674

[101] Paragliola RM, Papi G, Pontecorvi A, Corsello SM (2017) Treatment with synthetic glucocorticoids and the hypothalamus-pituitary-adrenal Axis. Int J Mol Sci 18(10):2201. 10.3390/ijms18102201 29053578 10.3390/ijms18102201 PMC5666882

[102] Mushtaq T, Shakur F, Wales JK, Wright NP (2008) Reliability of the low dose synacthen test in children undergoing pituitary function testing. J Pediatr Endocrinol Metab 21(12):1129–1132. 10.1515/jpem.2008.21.12.1129 19189685 10.1515/jpem.2008.21.12.1129

[103] Guerrero Pérez F, Marengo AP, Villabona Artero C (2017) The unresolved riddle of glucocorticoid withdrawal. J Endocrinol Invest 40(11):1175–1181. 10.1007/s40618-017-0691-1 28528436 10.1007/s40618-017-0691-1

[104] Jespersen S, Nygaard B, Kristensen LØ (2015) Methylprednisolone Pulse Treatment of Graves’ Ophthalmopathy is not Associated with secondary adrenocortical insufficiency. Eur Thyroid J 4(4):222–225. 10.1159/000440834 26835424 10.1159/000440834 PMC4716422

[105] Schaik IN, Eftimov F, Doorn PA et al (2010) Pulsed high-dose dexamethasone versus standard prednisolone treatment for chronic inflammatory demyelinating polyradiculoneuropathy (PREDICT study): a double‐blind, randomised, controlled trial. Lancet Neurol 9(3):245–253. 10.1016/S1474-4422(10)70021-1 20133204 10.1016/S1474-4422(10)70021-1

[106] Bornstein SR, Allolio B, Arlt W et al (2016) Diagnosis and treatment of primary adrenal insufficiency: an endocrine Society Clinical Practice Guideline. J Clin Endocrinol Metab 101(2):364–389. 10.1210/jc.2015-1710 26760044 10.1210/jc.2015-1710 PMC4880116

[107] Maguire AM, Biesheuvel CJ, Ambler GR, Moore B, McLean M, Cowell CT (2008) Evaluation of adrenal function using the human corticotrophin-releasing hormone test, low dose Synacthen test and 9 am cortisol level in children and adolescents with central adrenal insufficiency. Clin Endocrinol (Oxf) 68(5):683–691. 10.1111/j.1365-2265.2007.03100.x 18070143 10.1111/j.1365-2265.2007.03100.x

[108] Sbardella E, Isidori AM, Woods CP, Argese N, Tomlinson JW, Shine B et al (2017) Baseline morning cortisol level as a predictor of pituitary-adrenal reserve: a comparison across three assays. Clin Endocrinol (Oxf) 86(2):177–184. 10.1111/cen.13232 27616279 10.1111/cen.13232

[109] Chanson P, Guignat L, Goichot B et al (2017) Adrenal insufficiency: screening methods and confirmation of diagnosis. Ann Endocrinol (Paris) 78(6):495–511. 10.1016/j.ando.2017.10.005 29174200 10.1016/j.ando.2017.10.005

[110] Fleseriu M, Hashim IA, Karavitaki N, Melmed S, Murad MH, Salvatori R, Samuels MH (2016) Hormonal replacement in hypopituitarism in adults: an endocrine Society Clinical Practice Guideline. J Clin Endocrinol Metab 101(11):3888–3921. 10.1210/jc.2016-2118 27736313 10.1210/jc.2016-2118

[111] Husebye ES, Pearce SH, Krone NP, Kämpe O (2021) Adrenal insufficiency. Lancet 397(10274):613–629. 10.1016/S0140-6736(21)00136-7 33484633 10.1016/S0140-6736(21)00136-7

[112] Kazlauskaite R, Evans AT, Villabona CV et al (2008) Corticotropin tests for hypothalamic-pituitary- adrenal insufficiency: a metaanalysis. J Clin Endocrinol Metab 93(11):4245–4253. 10.1210/jc.2008-0710 18697868 10.1210/jc.2008-0710

[113] Ospina NS, Al Nofal A, Bancos I, Javed A, Benkhadra K, Kapoor E, Lteif AN, Natt N, Murad MH (2016) ACTH Stimulation tests for the diagnosis of adrenal insufficiency: systematic review and Meta-analysis. J Clin Endocrinol Metab 101(2):427–434. 10.1210/jc.2015-1700 26649617 10.1210/jc.2015-1700

[114] Dökmetaş HS, Colak R, Keleştimur F, Selçuklu A, Unlühizarci K, Bayram F (2000) A comparison between the 1-microg adrenocorticotropin (ACTH) test, the short ACTH (250 microg) test, and the insulin tolerance test in the assessment of hypothalamo-pituitary-adrenal axis immediately after pituitary surgery. J Clin Endocrinol Metab 85(10):3713–3719. doi: 10.1210/jcem.85.10.6879. Erratum in: J Clin Endocrinol Metab 2001;86(7):3085 10.1210/jcem.85.10.6879 11061529

[115] Wade M, Baid S, Calis K, Raff H, Sinaii N, Nieman L (2010) Technical details influence the diagnostic accuracy of the 1 microg ACTH stimulation test. Eur J Endocrinol 162(1):109–113. 10.1530/EJE-09-0746 19797501 10.1530/EJE-09-0746 PMC2941345

[116] Ng SM, Agwu JC, Dwan K (2016) A systematic review and meta-analysis of synacthen tests for assessing hypothalamic-pituitary-adrenal insufficiency in children. Arch Dis Child 101(9):847–853. 10.1136/archdischild-2015-308925 26951687 10.1136/archdischild-2015-308925

[117] Hawley JM, Owen LJ, Lockhart SJ et al (2016) Serum cortisol: an Up-To-Date Assessment of Routine Assay performance. Clin Chem 62(9):1220–1229. 10.1373/clinchem.2016.255034 27440512 10.1373/clinchem.2016.255034

[118] Cortez S, Arbeláez AM, Wallendorf M, McNerney K (2023) Peak serum cortisol cutoffs to diagnose adrenal insufficiency across different cortisol assays in children. J Clin Res Pediatr Endocrinol 15(4):375–379. 10.4274/jcrpe.galenos.2023.2023-2-3 37218135 10.4274/jcrpe.galenos.2023.2023-2-3 PMC10683551

[119] Kazlauskaite R, Maghnie M (2010) Pitfalls in the diagnosis of central adrenal insufficiency in children. Endocr Dev 17:96–107. 10.1159/000262532 19955760 10.1159/000262532 PMC3959797

[120] Javorsky BR, Raff H, Carroll TB, Algeciras-Schimnich A, Singh RJ, Colón-Franco JM, Findling JW (2021) New Cutoffs for the Biochemical Diagnosis of Adrenal Insufficiency after ACTH Stimulation using specific cortisol assays. J Endocr Soc 5(4):bvab022. 10.1210/jendso/bvab022 33768189 10.1210/jendso/bvab022 PMC7975762

[121] Gill H, Barrowman N, Webster R, Ahmet A (2019) Evaluating the low-dose ACTH stimulation test in children: Ideal Times for Cortisol Measurement. J Clin Endocrinol Metab 104(10):4587–4593. 10.1210/jc.2019-00295 31219559 10.1210/jc.2019-00295

[122] Park J, Didi M, Blair J (2016) The diagnosis and treatment of adrenal insufficiency during childhood and adolescence. Arch Dis Child 101(9):860–865. 10.1136/archdischild-2015-308799 27083756 10.1136/archdischild-2015-308799

[123] Debono M, Elder CJ, Lewis J et al (2023) Home waking salivary cortisone to screen for adrenal insufficiency. NEJM Evid 2(2):EVIDoa2200182. 10.1056/EVIDoa2200182 38320034 10.1056/EVIDoa2200182

[124] de Weerth C, Zijl RH, Buitelaar JK (2003) Development of cortisol circadian rhythm in infancy. Early Hum Dev 73(1–2):39–52. 10.1016/s0378-3782(03)00074-4 12932892 10.1016/s0378-3782(03)00074-4

[125] Mushtaq T, Ali SR, Boulos N et al (2023) Emergency and perioperative management of adrenal insufficiency in children and young people: British Society for Paediatric Endocrinology and Diabetes consensus guidance. Arch Dis Child 108(11):871–878. 10.1136/archdischild-2022-325156 37045585 10.1136/archdischild-2022-325156 PMC10646833

[126] Nowotny H, Ahmed SF, Bensing S et al (2021) Therapy options for adrenal insufficiency and recommendations for the management of adrenal crisis. Endocrine 71(3):586–594. 10.1007/s12020-021-02649-6 33661460 10.1007/s12020-021-02649-6 PMC7929907

[127] Rushworth RL, Torpy DJ, Stratakis CA, Falhammar H (2018) Adrenal crises in children: perspectives and research directions. Horm Res Paediatr 89(5):341–351. 10.1159/000481660 29874655 10.1159/000481660

[128] Mitchell AL, Napier C, Asam M et al (2014) Saving lives of in-patients with adrenal insufficiency: implementation of an alert scheme within the Newcastle-upon-Tyne hospitals e-Prescribing platform. Clin Endocrinol (Oxf) 81(6):937–938. 10.1111/cen.12457 24712680 10.1111/cen.12457

